# Supplementary material for: Ribosome Biogenesis Serves as a Therapeutic Target for Treating Endometriosis and the Associated Complications
Source: Biomedicines. 2022 Jan 17;10(1):185. doi: 10.3390/biomedicines10010185 (PMC8774031; doi:10.3390/biomedicines10010185)
Supplement: Supplementary file 1 [file biomedicines-10-00185-s001.zip › biomedicines-1540705-supplementary.pdf]

Table S1. Information for antibodies used in this study

**Antibodies for flow cytometry**					
Marker	*Conjugate*	*Cell type*	*Dilution*	*Brand*	*Cat. No.*
CD3	PE/Dazzle™ (594nm)	T-cell	1/80	BioLegend	100347
F4/80	PECy7 (561nm)	Macrophage (LpM)	1/200	BioLegend	123114
Ly6C	PE (488nm)	Monocyte	1/400	BD	560592
MHCII	APC-Cy7 (633nm)	Macrophage (SpM)	1/400	BioLegend	107627
Ly6G	PE-Texas Red (561nm)	Neutrophil	1/200	BD	562700
CD45.2	PerCP-Cy5.5 (488nm)	Lymphocyte	1/200	BioLegend	109827
CD3	APC (633nm)	T cell	1/200	BioLegend	100236
CD19	APC (633nm)	B cell	1/200	BioLegend	152410
CD49b	APC (633nm)	NK cell	1/200	BD	560628
Siglec F	APC (633nm)	Eosinophil	1/200	Miltenyi	130-102-241
FVS660	APC (633nm)	Dead cell	1/1000	BD	564405
**Antibodies for IHC study**					
Marker	*Conjugate*	*Ab type*	*Dilution*	*Brand*	*Cat. No.*
Vimentin	N/A	Primary	1：50	Cell	5741
Cytokeratin	N/A	Primary	1：50	Abcam	Ab93279
Novolink Polymer Detection Systems	HRP	Secondary (for anti-rabbit)	1:1	Leica	RE7140-K
**Antibodies for IF study**					
Marker	*Conjugate*	*Ab type*	*Dilution*	*Brand*	*Cat. No.*
PGP9.5	N/A	Primary	1：100	Millipore	AB1761-I
CD31	N/A	Primary	1：2000	Novus	NB100-1642
Goat anti Rabbit IgG	Rodamine	Secondary	1：1000	ABgent	ASR1424
Goat anti Rat IgG	FITC	Secondary	1：500	Arigo	ARG23755

Table S2. Primer sequences for real-time qPCR study[a]

Gene	*Forward primer (5' to 3')*	*Reverse primer (5' to 3')*	*UPL probe*[b]
COX-2	GATGCTCTTCCGAGCTGTG	GGATTGGAACAGCAAGGATTT	45
IL-6	TCTAATTCATATCTTCAACCAAGAGG	TGGTCCTTAGCCACTCCTTC	78
IL-1β	AGTTGACGGACCCCAAAAG	TTTGAAGCTGGATGCTCTCAT	26
TNF-α	CTGTAGCCCACGTCGTAGC	TTTGAGATCCATGCCGTTG	25

[a]Those primers were designed for mouse inflammatory cytokines.

[b]UPL: Universal Probe Library from Roche Applied Science (Penzberg, Germany)
